# Gene therapy for cancer through adenovirus vector-mediated expression of the Ad5 early region gene 1A based on loss of IGF2 imprinting

**DOI:** 10.3892/or.2013.2646

**Published:** 2013-07-30

**Authors:** YUQIN PAN, BANGSHUN HE, ZHANG LIRONG, ZHENLIN NIE, LIPING CHEN, LING GU, ANDREW R. HOFFMAN, SHUKUI WANG, JIFAN HU

**Affiliations:** 1 Central Laboratory of Nanjing First Hospital, Nanjing Medical University, Nanjing, Jiangsu 210012, P.R. China; 2 Department of Life Sciences, Nanjing Normal University, Nanjing, Jiangsu 210046, P.R. China; 3 Department of Medicine, Stanford University Medical School, Palo Alto, CA 94304, USA; 4 VA Palo Alto Health Care System, Palo Alto, CA 94304, USA

**Keywords:** genomic imprinting, insulin-like growth factor 2 gene, CTCF, adenoviral vector, gene therapy

## Abstract

Loss of (genomic) imprinting (LOI) of the insulin-like growth factor 2 gene (*IGF2*) is a common epigenetic abnormality in many human cancers. *IGF2* imprinting is regulated by differentially methylated domains (DMD) in the imprinting control region that is located between *IGF2* and *H19* on human chromosome 11. In the present study, combined expression of adenoviral vectors (Ad-EGFP and Ad-E1A) driven by H19 enhancer-DMD-H19 promoter complex was investigated and their effects on the tumor growth were assessed *in vitro* and *in vivo*. When infected with Ad-EGFP, the cancer cell lines with the LOI, such as HRT-18 and HT-29 cells, had the expression of the EGFP protein, whereas three cancer cell lines with the maintenance of imprinting (MOI) (HCT-116, MCF-7 and GES-1) had weak expression of EGFP. Furthermore, the expressed Ad-E1A significantly decreased cell viability and induced cell apoptosis only in HRT-18 and HT-29 cells *in vitro*, and effectively suppressed tumor development in HRT-18 and HT-29 xenograft in nude mice. It is concluded that this gene therapy vector is effective in the suppression of the growth of human colon cancer cells *in vitro* and *in vivo*, and that cancer gene therapy based on loss of *IGF2* imprinting may prove to be a novel therapeutic option.

## Introduction

Genomic imprinting refers to the epigenetic modification of a genome without the alteration in the DNA sequence, and imprinted genes are expressed in a parent-of-origin dependent manner. Insulin-like growth factor 2 gene (IGF2) is a paternally expressed imprinted gene, but IGF2 imprinting is lost in a host of human neoplasm, leading to increased IGF2 expression (1,2). IGF2 imprinting is regulated by either an imprinting control region (ICR) or the differentially methylated domain (DMD) of the IGF2/H19 imprinting domain located on chromosome 7 (3,4). It acts by regulating interactions between the H19 and IGF2 promoters and their shared enhancers, which lie downstream of the H19. Deletion of the ICR/DMD results in loss of imprinting (LOI) of the IGF2 gene (5). Proper imprinting of IGF2 requires that the ICR/DMD is methylated on the paternal allele and unmethylated on the maternal allele. The ICR located between the IGF2 and H19 contains four CTCF binding sites that are differentially methylated according to their parental origin (6,7). When unmethylated, such regions are bound by the insulator protein CCCTC-binding factor (CTCF) (6–8). As an insulator of transcription, CTCF is a suitable candidate as one of the putative imprinting factors. When CTCF levels are diminished by RNA interference (RNAi) in mouse fibroblasts, IGF2 imprinting is partially lost (9). CTCF binding to the DMD is critical in the establishment of IGF2 imprinting in the mouse. Deletion of the locus containing the DMD led to biallelic expression of IGF2 (5,10). Mutation of each CTCF binding site in the DMD also altered IGF2 imprinting (11). Using a transgenic RNAi-based approach to generate oocytes with reduced CTCF expression, Fedoriw *et al* found that CTCF protected the ICR from *de novo* methylation during oocyte growth and was required for normal preimplantation development (12).

A number of studies have observed loss of imprinting (LOI) of IGF2 in both tumor tissues and tumor cell lines. For example, the LOI of IGF2 was identified in 8 out of 14 different tumor cell lines, possibly as the result of inactivation or mutation of CTCF complex (13), suggesting that the LOI of IGF2 is associated with the loss of activity of CTCF due to its inactivation or mutation in tumor cells (known as LOI of IGF2), and that an abnormal tumor epigenotype could be corrected by *in vitro* reprogramming.

To obtain sufficient antitumoral effect, it is critical to deliver therapeutic genes efficiently into target cancer cells (14,15). Adenovirus (Ad) vectors can infect a broad range of human cells with high efficiency and achieve high levels of transgene expression. Moreover, the Ad viral genome is genetically stable and the inserted foreign genes are generally maintained without change through successive rounds of viral replication (16). These features make Ad vectors attractive in gene therapy. As a therapeutic gene, we chose the Ad5 early region gene 1A (E1A), whose products are the first adenoviral proteins produced upon infection and function primarily as the activation of transcription of the other viral early gene products (17). The E1A proteins affect host cell growth that is thought to allow more efficient production of viral progeny. In addition, E1A may induce apoptosis (18), and E1A may enhance sensitivity to chemotherapeutics and radiation. Of note, normal cells appear to be unaffected by the E1A protein (19).

The present study constructed an adenoviral vector for tumor cell targeted therapy based on the principle of LOI of IGF2 in tumor cells. The therapeutic potential of a vector carrying the E1A gene driven by enhancer-DMD-H19 promoter complex was tested both in HRT-18 and HT-29 human ileocolon cells (LOI of IGF2), HCT-116 human ileocolon cells (MOI of IGF2) and MCF-7 human breast cancer cells (MOI of IGF2) and GES-1 human gastric epithelial cells (MOI of IGF2). The constructed vector carrying the E1A gene was found to infect and replicate selectively with high efficiency, and had an effective antitumor activity in HRT-18 and HT-29 human colon cells as well as xenografts in nude BALB/c mice *in vivo*.

## Materials and methods

### Cell lines and cell culture

Human colon cancer cells (HRT-18 and HT-29) with loss of imprinting of IGF2 gene (LOI) were obtained from the American Type Culture Collection (ATCC, Manassas, VA, USA). The human colon cancer cells (HCT-116), breast cancer cells (MCF-7) and human gastric epithelial cells (GES-1) which carried maintenance of imprinting of IGF2 gene (MOI each) were also obtained from the ATCC. All cells were maintained in DMEM (HyClone, Logan, UT, USA) supplemented with 10% fetal bovine serum (FBS; Hyclone) and 100 mg/ml penicillin, and 50 mg/ml streptomycin, at 37ºC under humidified conditions of 95% air and 5% CO_2_.

### Plasmid construction and virus packaging

The original adenoviral shuttle plasmid used in this study was pDC312. The mouse H19 enhancer exon 1 (258 bp) and the mouse H19 enhancer exon2 (360 bp) were amplified by PCR from mouse genomic DNA, and the mouse DMD exon 1–2 (429 bp), DMD exon 3 (207 bp) and DMD exon 4 (156 bp) were also amplified by PCR from mouse genomic DNA, and then they were linked to a single fragment by PCR. Subsequently, the enhancer-DMD (1376 bp) was cloned into the plasmid pDC312 using restriction endonuclease *Xba*I and *Sal*I. The mouse H19 promoter (302 bp) was amplified by PCR from mouse genomic DNA using the upper primer 5′-GCAAGCTT CCACCGTTCTATGAAGGGCTTC-3′ (5′-primer for mouse H19 with *Sal*I) and lower primer 5′-AAGAATTCTCATCAG CGCCCATCTCTAGCC-3′ (5′ primer for mouse H19 with *Hin*dIII), cloned into the downstream of the enhancer-DMD using restriction endonuclease *Sal*I and *Hind*III. The E1A segment (1013 bp) was amplified by PCR from the pDC312 using the upper primer 5′-CCCAAGCTTGGGCCCTATG AGACATATTATCT-3′ (5′ primer for E1A with *Eco*RI) and lower primer 5′-CGCGGATCCCGCAATCACAGGTTTAC ACCTTA-3′ (5′ primer for E1A with *Bam*HI). The enhanced green fluorescent protein (EGFP) reporter gene from pEGFP-C1 vector (Clontech, Mountain View, CA, USA) and the E1A gene were inserted, respectively, into the downstream of the H19 promoter using restriction endonuclease *Eco*RI and *Bam*HI to construct pDC312-enhancer-DMD-H19-EGFP and pDC312-enhancer-DMD-H19-E1A. The product was confirmed by DNA sequencing. The plasmids carrying the target gene were transfected into HEK293 cells using liposome Lipofectamine™ 2000 (Invitrogen-Life Technologies, Carlsbad, CA, USA) together with the adenoviral vector Ad5. The culture solution was changed after 6 h, and the cytopathic effect (CPE) was observed continuously. CPE was found after 7–10 days. When the majority of the pathologically abnormal cells had come off the bottom of the culture flask, the abnormal cells and supernatant were collected, frozen and thawed at −80ºC/37ºC three times and centrifuged; the supernatant was then collected and, finally, two sets of adenoviruses were obtained: Ad-EGFP and Ad-E1A.

### Analysis of EGFP expression in the constructed plasmids

All cells were infected with Ad-EGFP (10 plaque forming units (PFU)/cell), respectively. EGFP expression was examined at 24, 48 and 72 h after infection using an Olympus microscope (Axioskop; Carl Zeiss Inc., Oberkochen, Germany) with a fluorescent filter set (excitation 450–490 nm).

### Analysis of the expression of hexon and E1A mRNA in virus-infected cells by real-time quantitative PCR

The hexon and E1A mRNA expression was determined by real-time quantitative PCR (RT-qPCR). All cells were infected with Ad-E1A (10 PFU/cell), respectively. Total RNA was extracted from the three cell lines with TRIzol (Invitrogen-Life Technologies) according to the manufacturer’s instructions. Following treatment with DNase I (Takara Biotechnology) at 37ºC for 30 min, RNA quantification was performed using spectrophotometry. The RNA (1 μg) was subsequently incubated with 1 μl of Oligo dT primer (50 μM), 1 μl of Random 6 mers (100 μM), 1 μl of PrimeScript™ RT Enzyme MixI, 4 μl of 5X PrimeScript™ Buffer and RNase Free dH_2_O, and first-strand cDNA synthesis was performed in a total volume of 20 μl. The primers used for hexon, E1A and β-actin are shown in Table I. The PCR reactions were performed in a LightCycler apparatus using real-time PCR Master Mix SYBR-Green I (Yotobo Biotech Co., Ltd., Osaka, Japan). Thermocycling was carried out in a final volume of 25 μl containing 1 μl of cDNA sample, 0.5 μl of the up-primer, 0.5 μl down-primer, 12.5 μl of SYBR-Green Real-time PCR Master Mix and 10.5 μl of dH_2_O. After 15 sec at 95ºC to denature the cDNA and to activate Taq DNA polymerase, the cycling conditions were: 40 cycles consisting of denaturation at 95ºC for 5 sec, annealing at 60ºC for 5 sec and extension at 72ºC for 30 sec. The Ct used in the real-time PCR quantification was defined as the PCR cycle number that crossed an arbitrarily chosen signal threshold in the log phase of the amplification curve. To verify the fold-change of E1A gene expression, calculated Ct values were normalized to Ct values of β-actin amplified from the same sample [ΔCt = Ct (E1A) − Ct (β-actin)], and the 2^−ΔΔCt^ method was used to calculate fold-change. Each sample had triplicates and all reactions were triplicated independently to ensure the reproducibility of the results.

### Analysis of E1A protein expression by western blot analysis

The E1A protein expression was evaluated by western blot analysis. Cells were harvested and lysed by three cycles of freeze/thaw at −80ºC. Total protein was separated by SDS-PAGE in 12% gels and transferred to a polyvinylidene difluoride (PVDF) membrane. The immunoblotting was performed as described elsewhere (20), and 5% skimmed milk powder (soluble in TBST buffer solution) was used at room temperature under sealed conditions for 1 h, with mouse anti-E1A primary antibodies (1:500) and rabbit anti-human β-actin primary antibodies (1:500) incubated at room temperature for 2 h, and then washed by vibrating with TBST solution. The proteins were visualized by ECL.

### Analysis of the cytotoxic effect of the virus by MTT and flow cytometry

The assay was based on the ability of viable cells to reduce MTT [3-(4,5-dimethylthiazol-2-yl)-2,5-diphenyl tetrazolium bromide; Sigma Aldrich, St. Louis, MO, USA] to insoluble colored formazan crystals. The cells were infected with adenoviruses increased multiplicity of infection (MOI) from 0 to 100 PFU/cell, and fresh medium was added at 5 h after infection and every 2 days thereafter. After 48, 72 and 96 h, MTT (5 mg/ml) was added to each well. After 4 h of incubation at 37ºC, the medium was removed, and 200 μl of DMSO (Sigma) were added into each well to dissolve the crystals. The absorbance was measured in a microplate reader at 490 nm (Bio-Rad Laboratories, Richmond, CA, USA). Quantitative evaluation of apoptosis was performed by flow cytometry after double staining with Annexin V fluorescein isothiocyanate (FITC) apoptosis detection kit, which allowed discrimination among early apoptotic (single Annexin V positive), and necrotic cells [double Annexin V/propidium iodide (PI) positive], which could differentiate cells that had lost membrane integrity (necrotic cells) from living cells by means of red staining of their nuclei with PI. Cells (1×10^6^ cells/well) were cultured in 6-well dishes, and then Ad-E1A were infected at 10 PFU/cell. Cell apoptosis was analyzed at 72 h after infection. Infection with Ad-EGFP (10 PFU/cell) served as a negative control.

### Treatment of tumor-bearing nude mice with the adenoviral vectors

Cells were trypsinized to a single cell suspension and resuspended in 10^9^ cells/100 μl PBS, then subcutaneously injected into the flank area of adult (8-week-old) athymic male nude mice (Harlan-Sprague-Dawley, Indianapolis, IN, USA). Two weeks after injection of HRT-8 or HT-29 cells, the developed tumors were measured in two dimensions. Then, Ad-E1A was injected into the left side and Ad-EGFP, serving as a viral vector control, was injected into the right side. Each adenoviral vector (a total dosage of 10^9^ PFU/mouse) was injected into a growing tumor from three directions on 3 successive days, and the tumor volume was observed for 28 days. Tumor dimensions were measured, and the tumor volume was calculated according to the formula (width)^2^ × length × 0.5.

### TUNEL assay

Apoptosis in tumor cells was detected by terminal deoxynucleotidyl transferase (TdT)-mediated dUTP nick-end labeling (TUNEL) assay. It was performed using *In Situ* Cell Death Detection kit (Roche, Mannheim, Germany) as per the manufacturer’s protocol. Sections were fixed in 30% formalin and rinsed. The sections were then incubated in 3% H_2_O_2_, permeabilized with 0.5% Triton X-100, rinsed again and incubated in the TUNEL reaction mixture. The sections were rinsed and visualized using Converter-POD with diaminobenzidine (DAB). Harris hematoxylin was used for counterstaining. The slides were air-dried at room temperature and cover slips were mounted using Permount. The number of TUNEL-positive cells was counted in six fields (at ×400 magnification of light microscope) selected at random, and the apoptosis index for each field was calculated as the percent of TUNEL-positive cells relative to the total.

### Statistical analysis

Experimental data are presented as the means ± SD (standard deviation) and assessed by the Student’s t-test and one-way ANOVA at a significance level of P<0.05.

## Results

### IGF2 imprinting

We measured the expression of IGF2 in selected human cell lines by RT-PCR, and three polymorphic restriction enzymes (*Apa*I, *Alu*I and *Hha*I) in the last exon for IGF2 were used to analyze allele-specific expression. As shown in Table II, human colon cancer cells (HRT-18 and HT-29) were with loss of imprinting of IGF2 gene (LOI), and the human colon cancer cells (HCT-116), breast cancer cells (MCF-7) and human gastric epithelial cells (GES-1) were maintenance of imprinting of IGF2 gene (MOI each).

### Verification of the recombinant plasmid and packaging of adenovirus

The recombinant plasmid pDC-312-enhance-DMD was digested by restriction endonuclease *Xba*I and *Sal*I to verify that the recombinant plasmid contained the enhancer-DMD fragment (1376 bp). The recombinant plasmid pDC-312-enhancer-DMD-H19 was digested by restriction endonuclease *Sal*I and *Hin*dIII to verify that the recombinant plasmid contained the H19 fragment (302 bp). The recombinant plasmid pDC-312-enhancer-DMD-H19-E1A and pDC-312-enhancer-DMD-H19-EGFP were digested by restriction endonuclease *Hin*dIII and *Bam*HI, to verify that the recombinant plasmid contained the E1A fragment (1013 bp) and EGFP fragment (718 bp), respectively. We confirmed that the recombinant plasmid had been constructed successfully. The plasmids pDC312-enhancer-DMD-H19-EGFP and pDC312-enhancer-DMD-H19-E1A were then transfected into HEK293 cells, respectively. After 8 days, the cells showed significant CPE phenomenon, and we obtained two sets of adenoviruses, i.e., Ad-EGFP and Ad-E1A.

### Analysis of selective expression of adenoviruses in the different tumor cells

**Figure 1:**
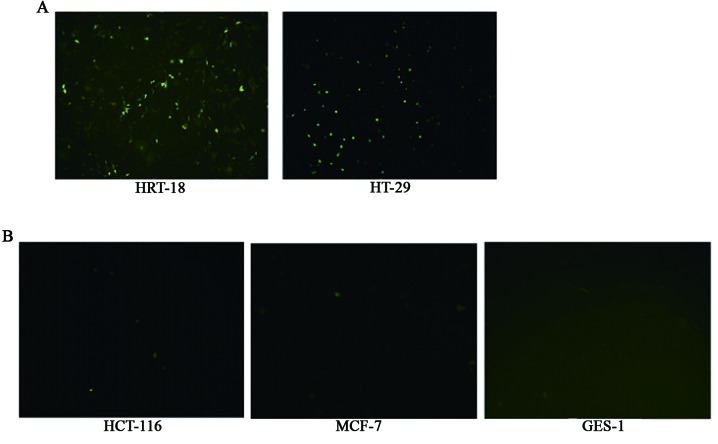
EGFP expression in the five cell lines infected with recombinant adenoviral vectors. (A) Infection with Ad-EGFP (10 PFU/cell) 24 h induced expression of EGFP in HRT-18 and HT-29 cells. (B) Microscope images show that there is only weak expression of EGFP in HCT-116, MCF-7 and GES-1 cells infected with Ad-EGFP (10 PFU/cell) at 48 h.

At 24 h after infection with Ad-EGFP (10 PFU/cell), the expression of EGFP protein was seen to be positive in the HRT-18 and HT-29 tumor cell lines, but negative in the other three types of cells (HCT-116, MCF-7 and GES-1). After 48 h, weak expression could be observed in the other three groups of cells (HCT-116, MCF-7 and GES-1), and the increase in the amount of the adenovirus or the infection time did not change the expression (Fig. 1).

### Levels and duration of the hexon gene expression in the different tumor cells infected with recombinant adenoviral vectors

**Figure 2:**
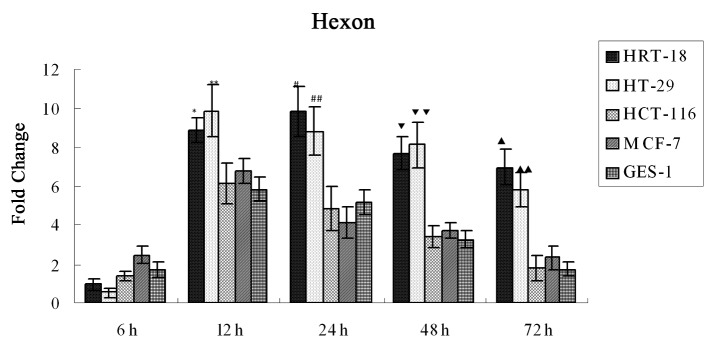
Analysis of the expression of hexon mRNA in infected cell lines. Real-time RT-PCR for hexon mRNA or β-actin (as an endogenous control for normalization) was performed and comparative quantification of hexon mRNA expression levels was carried out. The bars show the expression in relationship to HRT-18 cells after infecting with Ad-E1A 6 h (set to 1) as means ± SD of 3 separate experiments. Graphs are representative of 3 separate experiments. ^*^P<0.05, ^**^P<0.05 vs. expression level in MOI cells (HCT-116, MCF-7 and GES-1) at 12 h. ^#^P<0.01, ^##^P<0.01 vs. expression level in MOI cells at 24 h. ^▼^P<0.01, ^▼▼^P<0.01 vs. expression level in MOI cells at 48 h. ^▲^P<0.01, ^▲▲^P<0.01 vs. expression level in MOI cells at 72 h.

The expression of hexon mRNA was determined by RT-qPCR at various time-points after infection. The present study demonstrated that hexon expression levels in LOI cell lines (HRT-18 and HT-29) after infecting with Ad-E1A at 6, 12, 24, 48 and 72 h were significantly higher compared to hexon mRNA levels obtained with Ad-E1A in MOI cell lines (HCT-116, MCF-7 and GES-1) (P<0.05; P<0.01) (Fig. 2).

### E1A mRNA transcript and protein expression

**Figure 3:**
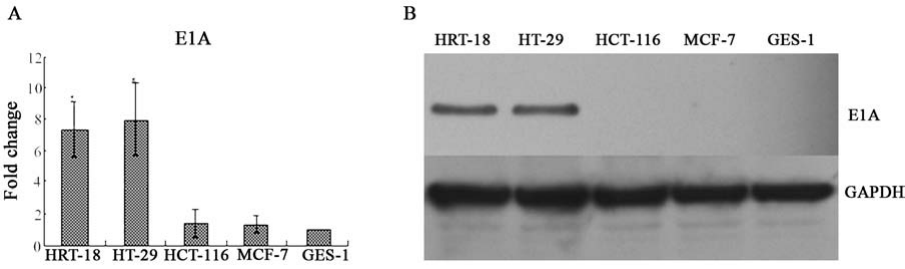
Analysis of the expression of E1A mRNA and protein in infected cell lines. (A) The expression of E1A and an internal control β-actin was evaluated by RT-qPCR after infecting with Ad-E1A (10 PFU/cell) 24 h. The bars show the expression in relationship to the GES-1 group (set to 1) as means ± SD of 3 separate experiments. E1A mRNA expression increased 7.36-fold in the HRT-18 group (^*^P<0.01), 7.94-fold in the HT-29 group (^*^P<0.01), 1.34-fold in the HCT-116 group (P>0.05) and 1.25-fold in the MCF-7 group (P>0.05). Graphs are representative of 3 separate experiments. (B) The expression of E1A and an internal control GAPDH was evaluated by western blot analysis after infecting with Ad-E1A (10 PFU/cell) 48 h. DT-A protein was positive in the HRT-18 and HT-29 cell lines, and negative in the HCT-116, MCF-7 and GES-1 cell lines.

The expression of E1A mRNA was determined by RT-qPCR at 24 h after infection, and the E1A protein expression was determined by western blot analysis at 48 h after infection. The present study demonstrated that the expression of E1A mRNA in the HRT-18 cells was 7.36-fold increased compared with that in the GES-1 group (P<0.01), and the expression of E1A mRNA in the HT-29 group was 7.94-fold increased compared with that in the GES-1 group (P<0.01). The expression of E1A mRNA in the HCT-116 group was 1.34-fold increased compared with that in the GES-1 group (P>0.05), and the expression of E1A mRNA in MCF-7 was 1.25-fold increased compared with that in the GES-1 group (P>0.05) (Fig. 3A). The E1A protein was positive in the HRT-18 and HT-29 tumor cell lines, and negative in HCT-116, GES-1 and MCF-7 cells (Fig. 3B).

### Cytotoxic effect and apoptosis induced by the adenoviruses

**Figure 4:**
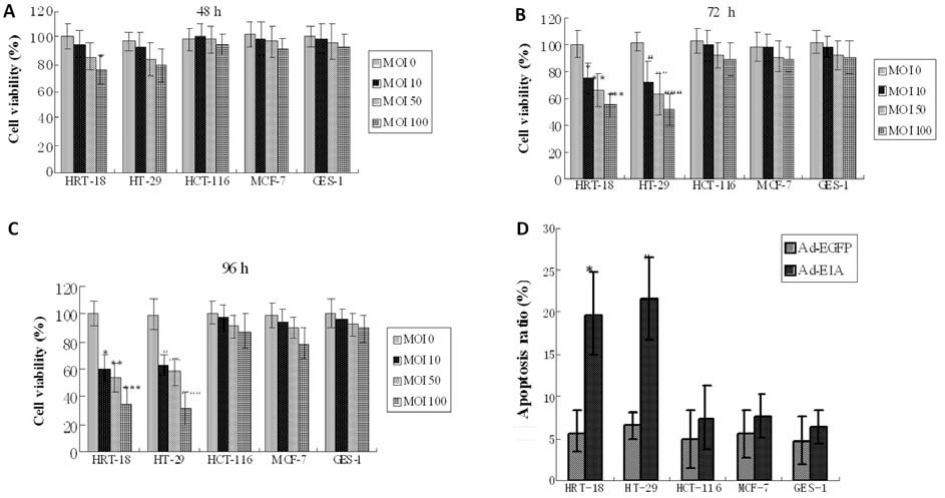
*In vitro* cytotoxic effect of adenoviral vectors carrying the E1A gene. (A–C) Cell viability was determined by MTT assay 48, 72 and 96 h after infecting with increased MOI from 0 to 100 PFU/cell in five cell lines. The cell viability in HCT-116, MCF-7 and GES-1 cells infected with Ad-E1A (0–10 PFU/cell) showed no significant increase compared with the control group (P>0.05), but minimal cytotoxicity was seen when infected with Ad-E1A (50–100 PFU/cell) at 48, 72 and 96 h (P>0.05). The cell viability in LOI cells (HRT-18 and HT-29) infected with Ad-E1A (100 PFU/cell) showed a significant decrease compared with the control group at 48 h (^*^P<0.05; ^#^P<0.05). The cell viability in LOI cells infected with Ad-E1A (10–100 PFU/cell) showed a significant decrease compared with the control group at 72 h (^*^P<0.05, ^**^P<0.05, ^***^P<0.01; ^#^P<0.05, ^##^P<0.05, ^###^P<0.01). The cell viability in LOI cells infected with Ad-E1A (10–100 PFU/cell) showed a significant decrease compared with the control group at 96 h (^*^P<0.01, ^**^P<0.01, ^***^P<0.01; ^#^P<0.01, ^##^P<0.01, ^###^P<0.01). Graphs are representative of 3 separate experiments. (D) The apoptosis of cells was investigated using flow cytometry analysis 72 h after infecting with Ad-E1A or Ad-EGFP (10 PFU/cell). The apoptotic ratio in MOI cell lines (HCT-116, MCF-7 and GES-1) infected with Ad-E1A showed no significant increase compared with the control group (P>0.05), but the apoptotic ratio in LOI cell lines (HRT-18 and HT-29) infected with Ad-E1A showed a significant increase compared with the control group (^*^P<0.01, ^#^P<0.01). Graphs are representative of 3 separate experiments.

Cell viability was assessed by MTT assay at 48, 72 and 96 h after infecting by recombinant adenoviral vectors increased MOI from 0 to 100 PFU/cell. Infecting with Ad-EGFP served as negative control to evaluate the cytopathic effect of adenoviral infection itself. The cytotoxicity in HCT-116, MCF-7 and GES-1 cells infected with Ad-E1A (0–10 PFU/cell) 48, 72 and 96 h showed no significant increase compared with the control group (P>0.05), but minimal cytotoxicity was observed when infected with Ad-E1A (50–100 PFU/cell) after 72 and 96 h (P>0.05). The cell viability in HRT-18 and HT-29 cells infected with Ad-E1A (100 PFU/cell) 48, 72 and 96 h showed a significant decrease compared with the control group (P<0.05), and when infecting with Ad-E1A (10–50 PFU/cell) 72 and 96 h also resulted in evident growth inhibition in the LOI cells (HRT-18 and HT-29) compared with the control group (P<0.05) (Fig. 4A–C). To assess the influence of the E1A on cell apoptosis, we analyzed the apoptosis in the three types of cells by flow cytometry at 72 h after infection. Infection with Ad-EGFP served as negative control to evaluate the cytopathic effect of adenoviral infection itself. The apoptosis rate in the HRT-18 and HT-29 cells (~20%) infected with Ad-E1A (10 PFU/cell) was higher than that in the control group (~6%) (P<0.01), but the percentage of apoptosis in HCT-116, MCF-7 and GES-1 cells infected with Ad-E1A (10 PFU/cell) showed no significant increase compared with the control group (P>0.05) (Fig. 4D).

### In vivo tumor growth inhibition by adenoviruses

**Figure 5:**
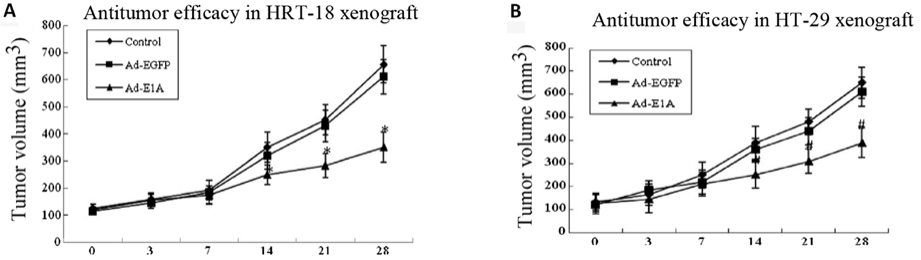
*In vivo* antitumor efficacy of adenoviral vectors carrying the E1A gene. (A and B) BALB/c mice were given subcutaneous implantation to establish tumor xenografts (HRT-18 and HT-29). Following the intratumoral injections with either the indicated adenoviruses (a total dosage of 10^9^ PFU/mouse), the tumor volume was observed for 28 days. The antitumor efficacy was marked in the Ad-E1A groups (^*^P<0.01 for Ad-E1A vs. control group after 14 days, ^#^P<0.01 for Ad-E1A vs. control group after 14 days).

We further confirmed the antitumor effect by injecting the indicated adenoviruses (a total dosage of 10^9^ PFU/mouse) into nude mice carrying HRT-18 and HT-29 tumor xenografts, respectively. The tumor growth was found to be similar and steady for all groups at the beginning, but after ~14 days the suppression of tumor growth was found to be strong and significantly superior in the Ad-E1A-treated groups, with the tumor inhibition rate of 30% in HRT-18 tumor xenografts compared to the buffer-control group (P<0.01) and 35% in HT-29 tumor xenografts compared to the buffer-control group (P<0.01). By comparison, no significant antitumor effect was observed in the Ad-EGFP group compared to the buffer-control group in HRT-18 and HT-29 tumor xenografts. (P>0.05) (Fig. 5).

### Immunohistology by TUNEL assay

**Figure 6:**
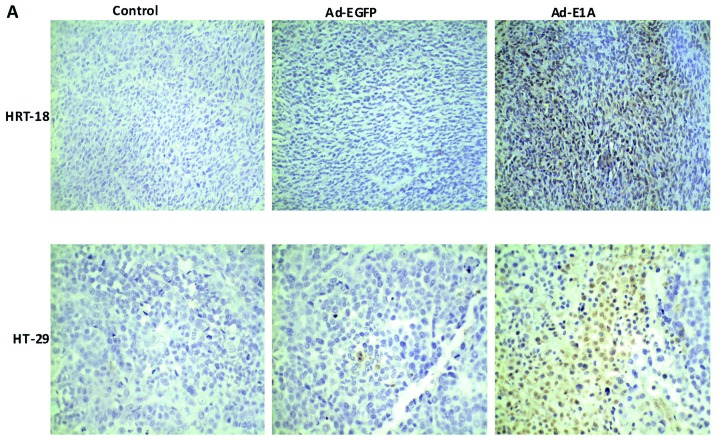

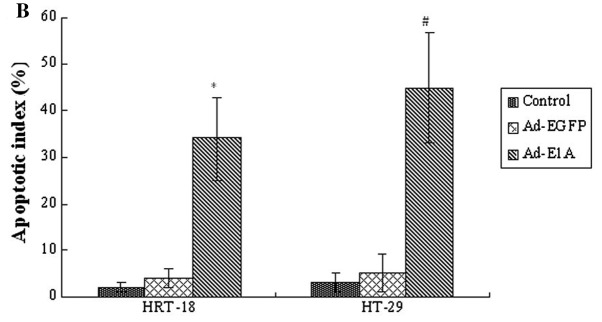
Tumor apoptosis was confirmed after therapy with recombinant adenoviral vectors by TUNEL assay. (A) Immunohistochemical staining for tumor apoptosis (TUNEL) after treatment with recombinant adenoviral vectors. Representative images at ×400 magnification. (B) Quantification of apoptotic index after therapy with recombinant adenoviral vectors. The number of TUNEL-positive cells counted from the randomly selected fields of each tumor section. (^*^P<0.01 vs. the PBS control, ^#^P<0.01 vs. the PBS control).

In order to further elucidate the mechanisms of the suppression of growth by Ad-E1A, the TUNEL assay was applied. Tumor apoptosis in HRT-18 and HT-29 tumor treatment nude mouse model was confirmed after treatment with Ad-E1A or Ad-EGFP or PBS by TUNEL assay. Nuclei of TUNEL-positive cells were stained brown and their shapes were condensed or segmented, whereas those of TUNEL-negative cells were stained blue by hematoxylin. Fig. 6A indicates a marked increase in apoptotic bodies in the Ad-E1A-treated mice tumor compared to Ad-EGFP-treated or PBS-treated groups. Apoptosis index was measured by the percentage of TUNEL-positive cells vs. total number of cells. The apoptotic indexes in HRT-18 of PBS, Ad-EGFP and Ad-E1A were 2±1, 4±2 and 34±9, respectively. The apoptotic indexes in HT-29 of PBS, Ad-EGFP and Ad-E1A were 3±2, 5±4 and 45±12, respectively. As mentioned above, the apoptotic index of HRT-18 and HT-29 tumors from mice treated with Ad-E1A was significantly higher than that from other groups (P<0.01) (Fig. 6B).

## Discussion

Genomic imprinting is essential for the normal growth and development of organisms (21). IGF2 is a paternally expressed imprinted gene, but IGF2 imprinting is lost in a host of human neoplasm, resulting in biallelic IGF2 expression and abnormally high IGF2 production. Earlier studies have shown that LOI of IGF2 is associated with somatic overgrowth and embryonal tumors and has been linked to more than 20 types of cancer in humans, including Wilms’ tumor and colorectal, lung, breast and prostate cancer (22). The current model of imprinting regulation of IGF2 is that binding of CTCF to the unmethylated maternal ICR/DMD protects it from *de novo* methylation and prevents downstream enhancers from activating IGF2, and CTCF is unable to bind the methylated paternal ICR/DMD that results in the expression of IGF2. Although it is now well established that the ICR/DMD acts as a CTCF-dependent insulator/enhancer blocker, the mechanism of insulation remains incomplete. Li *et al*(23) confirmed that the inactivation or mutation of the CTCF complex was closely related to the LOI of IGF2 in tumor cells by chromatin immunoprecipitation (CHIP) and chromosome conformation capture (3C) technique (23). Hu *et al* demonstrated that IGF2 aberrant epigenotype can be corrected by transferring nuclei from human tumor cells (LOI cells) into enucleated mouse and human fibroblasts (MOI cells) (13,24). The results demonstrate that an abnormal tumor epigenotype can be corrected by *in vitro* reprogramming, and suggest that LOI is associated with the inactivation or mutation of the CTCF complex; therefore, we designed our experiment based on the following mechanisms that in the MOI cells, the active CTCF can bind to the DMD, blocking the activity of enhancer and inhibiting the expression of the downstream genes, but that the inactive CTCF in the LOI cells cannot bind to the DMD, leading to increased expression of downstream genes as a result of the effect of the promoter activated by the enhancers. This is due to the fact that the enhancers physically interact with the IGF2 promoters on the paternal chromosome. However, interactions on the maternal chromosome remain unclear. Kurukuti *et al*(25) found maternal-specific silencing of IGF2 when the ICR/DMD interacts with a matrix attachment region and a differentially methylated region at the IGF2 locus to generate a tight loop around the IGF2 gene, thereby physically impeding IGF2 expression. By contrast, Yoon *et al* demonstrated that the ICR/DMD forms a transcriptionally unproductive association with enhancers and the inactive IGF2 promoters on the maternal chromosome, which leads to silencing of IGF2 (26).

In the present study, we examined the effects of enhancer-DMD-H19 promoter-driven E1A expression on the growth of tumor cells based on loss of imprinting of IGF2 gene. We constructed the expression vector to express E1A protein only seen in the cells that were loss of imprinting of IGF2 (inactive CTCF). Earlier studies from our group and others showed that normal cells were maintenance of IGF2 imprinting (active CTCF), we speculated that there was no cytotoxicity of E1A in normal cells. Firstly, the use of our expression system was identified by EGFP reporter assays, and data demonstrated that green fluorescence was positive in HRT-18 and HT-29 tumor cell lines that was loss of imprinting of IGF2, but only weak expression observed in HCT-116 cells, MCF-7 cells and GES-1 cells that were maintenance of imprinting of IGF2 regardless of the multiplicity of infection or the prolonged infection time. In the present study, expression of hexon genes was observed in the five infected cell lines at various time-points, suggesting that the recombinant adenovirus could infect LOI tumor cells effectively and gradually declined with time.

The E1A has been found to have an anticancer effect, and it is a tumor-suppressing gene commonly used in gene therapy. In the present study, the five types of the cells of different gene imprinting systems were infected with Ad-E1A and the cell viability was assessed by MTT. The results showed that there was no detectable cytotoxicity in HCT-116, MCF-7 and GES-1 cells when infected with Ad-E1A, and minimal cytotoxicity was seen when co-infected with Ad-E1A (50–100 PFU/cell) at 72 and 96 h. The results showed that E1A was very weak in inducing apoptosis in MOI of IGF2 cell lines (HCT-116, MCF-7 and GES-1), whereas it significantly induced apoptosis in LOI of IGF2 cell lines (HRT-18 and HT-29). Thus, we could observe imprinting-dependent induction of apoptosis in tumor cells infected with the E1A expression vectors. We also used the HRT-18 and HT-29 cell lines to establish a xenograft mouse model. The results showed a significant inhibition of tumor growth in Ad-E1A-treated groups compared to the control group. The apoptosis in HRT-18 animal model and HT-29 animal model were also confirmed using the immunohistochemical staining. The results showed that Ad-E1A increased the apoptotic index and suggested that Ad-E1A resulted in an increased apoptosis of tumor cells, thereby leading to the tumor growth inhibition, indicating that the E1A expression vector had a high therapeutic potential and was a promising candidate for colon cancer therapy in humans.

In conclusion, an IGF2 imprinting-based gene therapy vector has been developed that is effective in inhibiting the growth of human colon cancer cells *in vitro* and *in vivo*. This study provides, to the best of our knowledge, the first evidence of the use of this system for cancer gene therapy *in vitro* and *in vivo*. Since our data focused only on colon cancer therapy, further studies are required to explore the use of these vectors to gene therapy for other types of cancer that are loss of IGF2 imprinting, such as hepatoma, lung cancer, breast cancer, leiomyosarcoma, osteosarcoma, leukemia and Wilms’ tumor.

## Figures and Tables

**Table I:** The primers of hexon, E1A and β-actin for real-time PCR.

Target	Primer
hexon	Sense: 5′-ATGATGCCGCAGTGGTCTTA-3′ Antisense: 5′-GTCAAAGTACGTGGAAGCCAT-3′
E1A	Sense: 5′-CCCAAGCTTGGGCCCTATGAGACATATTATCT-3′ Antisense: 5′-CGCGGATCCCGCAATCACAGGTTTACACCTTA-3′
β-actin	Sense: 5′-CTGGAACGGTGAAGGTGACA-3′ Antisense: 5′-AAGGGACTTCCTGTAACAACGCA-3′

**Table II:** Genomic imprinting of IGF2 in selected human cell lines.

Cell lines	Tumor type	PREs	IGF2 imprinting
HRT-18	Colon cancer	*Hha*I	LOI
HT-29	Colon cancer	*Apa*I	LOI
HCT-116	Colon cancer	*Apa*I	MOI
MCF-7	Breast cancer	*Alu*I	MOI
GES-1	Human gastric epithelial cells	*Apa*I	MOI

PREs, polymorphic restriction enzymes used to distinguish the two parental alleles; LOI, loss of IGF2 imprinting (biallelic expression); MOI, maintenance of IGF2 imprinting (monoallelic expression).

## References

[1] Rainier S, Johnson LA, Dobry CJ, Ping AJ, Grundy PE, Feinberg AP (1993). Relaxation of imprinted genes in human cancer. Nature.

[2] Feinberg AP (1993). Genomic imprinting and gene activation in cancer. Nat Genet.

[3] Reik W, Constancia M, Dean W, Davies K, Bowden L, Murrell A, Feil R, Walter J, Kelsey G (2000). Igf2 imprinting in development and disease. Int J Dev Biol.

[4] Arney KL (2003). H19 and Igf2-enhancing the confusion?. Trends Genet.

[5] Thorvaldsen JL, Duran KL, Bartolomei MS (1998). Deletion of the H19 differentially methylated domain results in loss of imprinted expression of H19 and Igf2. Genes Dev.

[6] Hark AT, Schoenherr CJ, Katz DJ, Ingram RS, Levorse JM, Tilghman SM (2000). CTCF mediates methylation-sensitive enhancer-blocking activity at the H19/Igf2 locus. Nature.

[7] Bell AC, Felsenfeld G (2000). Methylation of a CTCF-dependent boundary controls imprinted expression of the Igf2 gene. Nature.

[8] Kanduri C, Pant V, Loukinov D (2000). Functional association of CTCF with the insulator upstream of the H19 gene is parent of origin-specific and methylation-sensitive. Curr Biol.

[9] Ling JQ, Li T, Hu JF (2006). CTCF mediates interchromosomal colocalization between Igf2/H19 and Wsb1/Nf1. Science.

[10] Leighton PA, Saam JR, Ingram RS, Stewart CL, Tilghman SM (1995). An enhancer deletion affects both H19 and Igf2 expression. Genes Dev.

[11] Schoenherr CJ, Levorse JM, Tilghman SM (2003). CTCF maintains differential methylation at the Igf2/H19 locus. Nat Genet.

[12] Fedoriw AM, Stein P, Svoboda P, Schultz RM, Bartolomei MS (2004). Transgenic RNAi reveals essential function for CTCF in H19 gene imprinting. Science.

[13] Chen HL, Li T, Qiu XW (2006). Correction of aberrant imprinting of IGF2 in human tumors by nuclear transfer-induced epigenetic reprogramming. EMBO J.

[14] Vile RG, Russell SJ, Lemoine NR (2000). Cancer gene therapy: hard lessons and new courses. Gene Ther.

[15] Gómez-Navarro J, Curiel DT, Douglas JT (1999). Gene therapy for cancer. Eur J Cancer.

[16] Robbins PD, Ghivizzani SC (1998). Viral vectors for gene therapy. Pharmacol Ther.

[17] Branton PE, Bayley ST, Graham FL (1985). Transformation by human adenoviruses. Biochim Biophys Acta.

[18] Radke JR, Siddiqui ZK, Miura TA, Routes JM, Cook JL (2008). E1A oncogene enhancement of caspase-2-mediated mitochondrial injury sensitizes cells to macrophage nitric oxide-induced apoptosis. J Immunol.

[19] Sánchez-Prieto R, Quintanilla M, Cano A (1996). Carcinoma cell lines become sensitive to DNA-damaging agents by the expression of the adenovirus E1A gene. Oncogene.

[20] Adler V, Yin Z, Fuchs SY (1999). Regulation of JNK signaling by GSTp. EMBO J.

[21] Szabó PE, Mann JR (1995). Biallelic expression of imprinted genes in the mouse germ line: implications for erasure, establishment, and mechanisms of genomic imprinting. Genes Dev.

[22] Kaneda A, Feinberg AP (2005). Loss of imprinting of IGF2: a common epigenetic modifier of intestinal tumor risk. Cancer Res.

[23] Li T, Hu JF, Qiu X (2008). CTCF regulates allelic expression of Igf2 by orchestrating a promoter-polycomb repressive complex 2 intrachromosomal loop. Mol Cell Biol.

[24] Hu JF, Vu TH, Hoffman AR (1996). Promoter-specific modulation of insulin-like growth factor II genomic imprinting by inhibitors of DNA methylation. J Biol Chem.

[25] Kurukuti S, Tiwari VK, Tavoosidana G (2006). CTCF binding at the H19 imprinting control region mediates maternally inherited higher-order chromatin conformation to restrict enhancer access to Igf2. Proc Natl Acad Sci USA.

[26] Yoon YS, Jeong S, Rong Q, Park KY, Chung JH, Pfeifer K (2007). Analysis of the H19ICR insulator. Mol Cell Biol.

